# Novel approaches for treating hypertension

**DOI:** 10.12688/f1000research.10117.1

**Published:** 2017-01-27

**Authors:** Andrew J. Freeman, Antony Vinh, Robert E. Widdop

**Affiliations:** 1 Department of Pharmacology and Monash Biomedicine Discovery Institute, Monash University, Clayton, Victoria 3800, Australia

**Keywords:** hypertension, cardiovascular disease, hypertension therapies, high blood pressure

## Abstract

Hypertension, or high blood pressure, is a prevalent yet modifiable risk factor for cardiovascular disease. While there are many effective treatments available to combat hypertension, patients often require at least two to three medications to control blood pressure, although there are patients who are resistant to such therapies. This short review will briefly update on recent clinical advances and potential emerging therapies and is intended for a cross-disciplinary readership.

## Introduction

Blood pressure is considered to be elevated at hypertensive levels when systolic blood pressure (SBP) is ≥140 mmHg and/or diastolic blood pressure is ≥90 mmHg. Hypertension is generally considered to be one of the strongest modifiable risk factors for cardiovascular disease. Its asymptomatic clinical presentation means that there is a long exposure time that contributes to cardiovascular complications and ultimately leads to a detrimental impact on global health.

Pharmacological treatment of hypertension decreases the likelihood of cardiovascular events such as heart attack, heart failure, and stroke occurring ^1– 3^, although blood pressure and associated cardiovascular diseases are still on the increase, particularly with our ageing population. The importance of blood pressure lowering can be seen with the outcomes from the recently published SPRINT trial ^4^. When SBP was intensively controlled to a target of <120 mmHg compared with the standard treatment target of <140 mmHg, intensive anti-hypertensive treatment resulted in ~25% reduction in primary composite outcome of myocardial infarction, acute coronary syndrome, stroke, heart failure, or cardiovascular death. Because of the striking findings, the trial was stopped ahead of time after a median follow-up of 3.3 years. While there is ongoing debate as to the applicability of such findings given the usually less rigorous clinical practice settings occurring in the general community, together with key patient exclusions (e.g. diabetes mellitus and stroke) ^5^, the high relevance of blood pressure control for organ protection is clearly evident. These findings are consistent with previous meta-analysis data on one million adults that demonstrated an association between increasing cardiovascular risk and blood pressure ^6^.

Many anti-hypertensive therapies are currently being used when lifestyle and behavioural changes are not sufficient. As our undergraduate students quickly learn, the “ABCD” of commonly prescribed anti-hypertensive agents (i.e. A=inhibitors of angiotensin [Ang] such as Ang-converting enzyme [ACE] inhibitors and Ang type I [AT _1_] receptor antagonists; B=β1-adrenoceptor antagonists; C=calcium channel antagonists; D=diuretics) are likely to be key initial choices. However, these drugs do not always adequately control blood pressure or are not appropriate in all hypertensive patients who usually exhibit various co-morbidities. Notwithstanding important issues such as noncompliance, it is still estimated that 10–15% of hypertensive patients are resistant to current treatment options, where blood pressure is uncontrolled with three or more different classes of anti-hypertensives, including a diuretic ^7, 8^. Therefore, the need for new treatment strategies to treat the multi-faceted nature of hypertension, including organ protection, is still an area of intensive research. This short review will discuss emerging novel approaches currently being investigated to treat hypertension.

## Combination therapies

Owing to the known clinical efficacy of inhibiting the renin-Ang system (RAS), one could speculate that additive multi-site therapy would result in maximal RAS blockade leading to enhanced anti-hypertensive effects and reduced end organ damage. Indeed, dual RAS blockade did show positive results from short-term studies using blood pressure and albuminuria as surrogate outcomes ^9– 11^, but subsequent longer-term trials measuring clinical outcomes have consistently shown that excessive RAS suppression causes adverse effects. For example, the ONTARGET ^12^, ALTITUDE ^13^, VA NEPHRON-D ^14^, and ATMOSPHERE ^15^ trials have confirmed, in a variety of high-risk patients with cardiovascular disease and/or diabetes or heart failure, that combination therapies that simultaneously inhibit a combination of renin, ACE, or AT _1_ receptors do not provide additional benefit and in fact exhibit adverse effects such as hypotension, hyperkalaemia, and renal dysfunction. The less favourable risk-benefit ratio of such dual RAS inhibition argues against this therapeutic strategy, and current hypertension guidelines do not recommend combined RAS inhibitor treatment ^1^.

Perhaps reflecting the need for rigorous blood pressure management, in an era of relatively few first-in-class anti-hypertensive agents, there have been numerous fixed double- and triple-dose combinations approved by the FDA this millennia, as recently reviewed ^16^.

## Re-purposing older drugs: mineralocorticoid receptor antagonists for treatment-resistant hypertension

There have been two mineralocorticoid receptor antagonists available for many decades. The second-generation compound eplerenone has reduced affinity for androgen and progesterone receptors compared with the first-generation antagonist spironolactone, but it is also less potent than spironolactone at blocking aldosterone receptors, hence the greater anti-hypertensive potency exhibited by spironolactone ^17^. Just as the RALES trial ^18^ led to a resurgence in the use of spironolactone and later eplerenone for the treatment of severe heart failure, there is renewed interest in the use of mineralocorticoid receptor antagonists for treatment-resistant hypertension (TRH), if this is caused by aldosterone breakthrough in patients already being treated with an ACE inhibitor or an AT _1_ receptor antagonist, leading to sodium retention ^17^.

While there have been a number of short-term (4–24 weeks’ duration) placebo-controlled trials that have shown beneficial effects of spironolactone in TRH ^17^, the strongest evidence for the use of spironolactone in TRH has recently been obtained from the PATHWAY-2 trial ^19^. All patients who were already receiving A+C+D medications were randomised to receive 12-week sequential treatments of placebo, spironolactone, the α _1_-adrenoceptor antagonist doxazosin, and the β _1_-adrenoceptor antagonist bisoprolol. In this study, spironolactone reduced home blood pressure recordings by approximately double that of other active treatment arms and showed for the first time a direct drug comparison in the same TRH patients. There was also a significant inverse correlation between plasma renin and blood pressure reduction by spironolactone, suggesting that sodium retention contributed to TRH. In this study ^19^ and earlier studies ^17^, there was a low incidence of adverse effects. While it has been argued that these results should influence treatment guidelines ^19^, it will be of great interest to determine long-term TRH outcomes in this particular cohort, including potential adverse outcomes, given the combined use of an ACE inhibitor or an AT _1_ receptor antagonist together with mineralocorticoid receptor blockade. Of interest, newer nonsteroidal mineralocorticoid receptor antagonists, such as finerenone, have been developed to target the heart and are being trialled in heart failure ^20^.

## Recent clinical advances

### Vasopeptidase inhibitors

Neprilysin (neutral endopeptidase 24.11) is an enzyme responsible for the breakdown of natriuretic peptides and has long been considered a target for hypertension. Indeed, inhibition of neprilysin increases natriuretic peptide levels, resulting in natriuresis, vasodilation, and functional inhibition of the RAS. However, any blood-pressure-lowering effect of neprilysin inhibition is offset, since this enzyme also degrades peptides such as endothelin-1 and Ang II that cause vasoconstriction. Indeed, combined neprilysin and ACE inhibition, achieved using omapatrilat, evoked greater anti-hypertensive effects than did the ACE inhibitor enalapril alone ^21^. However, the higher rate of angioedema noted by omapatrilat (most likely involving raised bradykinin levels) in large-scale heart failure trials has led to the discontinuation of this therapeutic strategy, despite clinical efficacy ^22^. Given that AT _1_ receptor antagonists, unlike ACE inhibitors, do not inhibit bradykinin metabolism, other combinations of RAS and neprilysin inhibition have been considered. Indeed, there is much interest in a new combination of equal proportions of the AT _1_ receptor antagonist valsartan and a neprilysin inhibitor sacubitril, known as LCZ696.

Clinical trial data for LCZ696 demonstrated significantly greater blood pressure reductions compared to valsartan in patients with mild-to-moderate hypertension ^23^. Anti-hypertensive efficacy of LCZ696 was confirmed in Asian populations with minimal safety and tolerability issues ^24^. The effect of LCZ696 has also been examined on biomarkers in heart failure patients with preserved ejection fraction (PARAMOUNT) ^25^ and on clinical outcomes in heart failure patients with reduced ejection fraction (PARADIGM-HF) ^26, 27^. The latter trial was terminated early since there was a 20% reduction in cardiac death compared with an ACE inhibitor. Interestingly, in all trials, blood pressure reductions were greater than the RAS inhibitor used as the comparator. The beneficial effects of the valsartan/sacubitril combination are described in detail elsewhere ^20, 28, 29^. Moreover, their striking effects in systolic heart failure patients and the prospects of benefits in future outcome trials for heart failure patients with preserved ejection fraction ^28, 29^ begs the question of the potential role of such vasopeptidase inhibition in resistant hypertension.

In addition, a dual vasopeptidase inhibitor called daglutril, combining neprilysin and endothelin-converting enzyme inhibition, has been developed and was reported to lower blood pressure in hypertensive type 2 diabetics who were already receiving losartan ^30^, although its current status is not clear.

### Anti-diabetic agents

The regulatory requirement to obtain robust cardiovascular safety data to approve new diabetes drugs has increased the number of trials focused on a lack of cardiovascular toxicity rather than examining longer-term studies on the effects of glucose lowering on cardiovascular outcome data ^31^. However, data are emerging showing that new anti-diabetic drugs can lower blood pressure, perhaps contributing to their therapeutic effects to improve glycaemic control and cardiovascular outcomes. The glucagon-like peptide 1 (GLP-1) analogue liraglutide reduced blood pressure in hypertensive diabetic patients ^32^. There was a small reduction in blood pressure in the recent LEADER trial, studying over 9,300 patients, in which liraglutide improved cardiovascular outcome (composite of death and non-fatal myocardial infarction or stroke) assessed over 3.5–5 years ^33^. Inhibitors of sodium-glucose cotransporter 2, such as empagliflozin, are another new class of anti-diabetic compound that impairs renal glucose reabsorption. In high-risk cardiovascular patients with type 2 diabetes, empagliflozin, added to standard care, lowered the rate of the primary composite cardiovascular outcome and of death from any cause ^34^ and reduced blood pressure versus placebo in patients with type 2 diabetes and hypertension ^35^. Thus, while these compounds are not strictly anti-hypertensive agents, even modest blood pressure reductions are likely to contribute to other cardiovascular protective mechanisms evoked by these novel anti-diabetic agents ^33, 36^.

## Emerging therapies

### Brain RAS inhibitors

Aminopeptidases are involved in the metabolism of Ang II into shorter Ang peptide fragments. Aminopeptidase A (APA) converts Ang II into Ang III by removing N-terminal aspartate, which can also activate AT _1_ or type 2 (AT _2_) receptors. Given the ubiquitous AT _1_ receptor expression, Ang II and Ang III both evoke predominant AT _1_ receptor-mediated peripheral vasoconstriction and pressor responses. Interestingly, preclinical studies have shown that centrally administered Ang III more effectively raised blood pressure than did Ang II (involving centrally mediated sympathetic nerve stimulation and vasopressin release) ^37, 38^. Such studies provided the rationale for inhibition of APA as a potential therapeutic strategy to treat hypertension ^38, 39^. RB150 is a dimer of EC33, which is a selective APA inhibitor that inhibits the conversion of Ang II to Ang III ^38, 39^. While the clinical efficacy of RB150, also known as QGC001, in the treatment of hypertension is yet to be evaluated, it was well tolerated in healthy male volunteers and did not significantly change heart rate or blood pressure ^40^. However, it remains to be seen if any centrally mediated anti-hypertensive effect of QGC001, due to decreased bioavailability of Ang III in the brain, is offset by an increase in peripheral Ang II accumulation and consequent vasoconstrictor potential in hypertensive patients. It is also less common nowadays for the development of centrally acting anti-hypertensive drugs because of central side effects.

### Protective arms of the renin-angiotensin system

In addition to the ACE/AT _1_ receptor arm of the RAS, there are two separate but related “protective” arms of the RAS, so called because their activation generally opposes AT _1_ receptor-mediated cardiovascular effects. These include the Ang III/AT _2_ receptor arm ^41, 42^ and the ACE2/Ang (1–7)/Mas receptor arm ^41, 43– 45^. Within the RAS, there are a number of bioactive Ang peptides in addition to the main effector Ang II. For example, Ang III can also exert more subtle depressor effects and natriuretic responses in preclinical studies that are mediated by AT _2_ receptor stimulation ^41, 42^. A number of selective AT _2_ receptor agonists have been developed ^45^, of which compound 21 (C21) has been the best studied in preclinical hypertension-related models. Indeed, selective AT _2_ receptor stimulation reduces hypertension-induced target organ damage, even in the absence of blood pressure reduction in most but not all instances ^42, 46^. The AT _2_ receptor field has been hampered by a lack of selective ligands, but there is great interest in the clinical effects of AT _2_ receptor activation and the potential for additive effects of AT _2_ receptor agonists with conventional RAS blockade. However, C21 has only entered phase I testing in healthy volunteers at this stage, so this field will be watched with interest.

ACE2 is a carboxypeptidase that differs to ACE in that it cleaves one (not two) amino acids from the C-terminal of peptides. In the context of RAS, Ang I and Ang II can be converted by ACE2 to Ang (1–9) and Ang (1–7), respectively. In particular, there is much preclinical evidence to indicate that Ang (1–7) can protect against hypertension-related target organ damage via another G-protein-coupled receptor known as the Mas receptor ^43, 44, 47^. Given the ability of ACE2 activation to facilitate conversion to the “cardioprotective” Ang (1–7) at the expense of (decreasing) levels of the substrate Ang II, research has focused on developing compounds that activate these RAS pathways. Indeed, there are a number of Mas receptor and AT _2_ receptor agonists ^20, 45^ that have not yet progressed beyond preclinical studies. Moreover, there are compounds that activate ACE2, such as diminazene aceturate (known as DIZI). Interestingly, this ACE2 activator, structurally related to xanthenone, is used clinically for the treatment of the parasitic disease African trypanosomiasis ^47^, so there is already translational potential. Similarly, recombinant ACE2 (rACE2) has been used in preclinical studies where it has been reported to lower blood pressure and exert cardioprotective effects ^43, 47^. Human rACE2 was tested in phase I studies in which single intravenous injections of varying doses were given to healthy subjects ^48^. Human rACE2 was well tolerated and increased plasma ACE2 without affecting blood pressure and evoked a prolonged, marked suppression of plasma Ang II levels ^48^. Clearly, additional clinical trial data are required to assess the veracity of this form of therapy.

## Renal denervation

Of the non-pharmacological methods being investigated to lower blood pressure ^20^, the effects of renal denervation are highly relevant and very topical. This interventional treatment was aimed at ablating the effects of augmented sympathetic drive to the kidney, which contributes to the pathogenesis of hypertension primarily owing to sympathetic activation and the release of renin. To this end, Symplicity HTN-1 ^49^ and Symplicity HTN-2 ^50^ trials used a minimally invasive catheter-based radiofrequency strategy to cause renal nerve denervation in an attempt to lower blood pressure in patients with TRH. These trials were very successful, with the reductions in blood pressure maintained for up to 3 years in many cases ^51, 52^. However, procedural concerns were raised about the study design (including lack of blinding or true resistant hypertension ^53^), which prompted the large multi-centre, sham-controlled, blinded Symplicity HTN-3 trial ^54^. Surprisingly, no significant blood pressure reductions were observed between the renal denervation and the sham groups after 6 months of follow-up ^54^, which has resulted in considerable controversy in the field of renal denervation. This discrepancy from earlier trials has been attributed to incomplete ablation after renal denervation, since later analysis revealed that complete renal denervation was rarely achieved in patients in Symplicity HTN-3 ^55^, most likely related to the inexperience of many trial operators and lack of procedural checks ^55, 56^. Recently, it has been cogently argued that the rationale for renal denervation for TRH remains valid ^56^. However, “smarter” renal denervation trials, with respect to design, location of ablation energy delivery, and testing of achieved denervation ^56^, are awaited with intense interest. The devil will be in the detail as to how this field, which holds great promise, progresses.

## Conclusions

Despite the effectiveness of the currently available anti-hypertensive medications, there is always a need for novel treatment strategies that are more effective in particular hypertensive patient groups and to provide additional resources to tackle TRH. Most of the newer drugs mentioned, including the emerging therapies, are likely to be useful for lowering blood pressure and/or limiting hypertension-related target organ damage.
